# Immucillins ImmA and ImmH Are Effective and Non-toxic in the Treatment of Experimental Visceral Leishmaniasis

**DOI:** 10.1371/journal.pntd.0004297

**Published:** 2015-12-23

**Authors:** Elisangela Oliveira Freitas, Dirlei Nico, Marcus Vinícius Alves-Silva, Alexandre Morrot, Keith Clinch, Gary B. Evans, Peter C. Tyler, Vern L. Schramm, Clarisa B. Palatnik-de-Sousa

**Affiliations:** 1 Departamento de Microbiologia Geral, Instituto de Microbiologia Paulo de Góes, Universidade Federal do Rio de Janeiro, Rio de Janeiro, Rio de Janeiro, Brazil; 2 Department of Biochemistry, Albert Einstein College of Medicine, Yeshiva University, New York, New York, United States; 3 Departamento de Imunologia, Instituto de Microbiologia Paulo de Góes, Universidade Federal do Rio de Janeiro, Rio de Janeiro, Rio de Janeiro, Brazil; 4 The Ferrier Research Institute, Victoria University of Wellington, Wellington, New Zealand; Northeastern University, UNITED STATES

## Abstract

**Background:**

Immucillins ImmA (IA), ImmH (IH) and SerMe-ImmH (SMIH) are synthetic deazapurine nucleoside analogues that inhibit *Leishmania (L*.*) infantum chagasi* and *Leishmania (L*.*) amazonensis* multiplication *in vitro* without macrophage toxicity. Immucillins are compared to the Glucantime standard drug in the chemotherapy of *Leishmania (L*.*) infantum chagasi* infection in mice and hamsters. These agents are tested for toxicity and immune system response.

**Methodology/Principal Findings:**

BALB/c mice were infected with 10^7^ amastigotes, treated with IA, IH, SMIH or Glucantime (2.5mg/kg/day) and monitored for clinical variables, parasite load, antibody levels and splenocyte IFN-γ, TNF-α, and IL-10 expression. Cytokines and CD4+, CD8+ and CD19+ lymphocyte frequencies were assessed in uninfected controls and in response to immucillins. Urea, creatinine, GOT and GPT levels were monitored in sera. Anti-*Leishmania*-specific IgG1 antibodies (anti-NH36) increased in untreated animals. IgG2a response, high levels of IFN-γ, TNF-α and lower levels of IL-10 were detected in mice treated with the immucillins and Glucantime. Immucillins permitted normal weight gain, prevented hepato-splenomegaly and cleared the parasite infection (85–89%) without renal and hepatic toxicity. Immucillins promoted 35% lower secretion of IFN-γ in uninfected controls than in infected mice. IA and IH increased the CD4+ T and CD19+ B cell frequencies. SMIH increased only the proportion of CD-19 B cells. IA and IH also cured infected hamsters with lower toxicity than Glucantime.

**Conclusions/Significance:**

Immucillins IA, IH and SMIH were effective in treating leishmaniasis in mice. In hamsters, IA and IH were also effective. The highest therapeutic efficacy was obtained with IA, possibly due to its induction of a TH1 immune response. Low immucillin doses were required and showed no toxicity. Our results disclose the potential use of IA and IH in the therapy of visceral leishmaniasis.

## Author Summary

The IA, IH, and SMIH immucillins are known to impair the replication of promastigotes of *L*. *(L*.*) infantum chagasi* and *L*. *(L*.*) amazonensis* imucillins *in vitro* and impair the replication of intracellular amastigotes of *L*. *(L*.*) infantum chagasi in vitro* with no toxicity for macrophages. IA and IH also inhibit the enzymatic activity of *Leishmania (L*.*) donovani* nucleoside hydrolase (NH36) one of the purine salvage enzymes in these purine auxotrophs. In this work, we compared the efficacies of IA, IH and SMIH to the standard drug Glucantime in the therapy of *L*. *(L*.*) infantum chagasi* infection of mice and hamsters. IA and IH, at low concentrations, cured mice and hamsters from visceral leishmaniasis (VL). Unlike treatment with Glucantime, immucillin therapy showed no toxicity. We demonstrate that treatment of IA and IH also affects the induction of the immune system, a factor that might also contribute to VL therapy. This study shows significant promise in the development of safer drugs for leishmaniasis therapy.

## Introduction

Visceral leishmaniasis (VL) is a neglected tropical disease [1] caused by *Leishmania (L*.*) donovani* in India and Central Africa, by *Leishmania (L*.*) infantum* in the Middle East, Central Asia, China and Mediterranean and by *Leishmania (L*.*) infantum chagasi* in America. Approximately 0.2–0.4 million new human cases [2] are registered annually, 90% of them in India, Bangladesh, Brazil, Nepal, Sudan, South Sudan and Ethiopia [1]. The infection is an anthroponoses in India, Central Africa and China and a canid zoonosis in the Mediterranean, China and Americas.

VL is the second most important vector-transmitted protozoa disease, second only to malaria [3] and an important opportunistic infection in patients with HIV [4]. Its main clinical signs in humans and dogs are hypergammaglobulinaemia, hepato-splenomegaly, malaise, anemia, cachexia, and progressive suppression of the cellular immune response. The VL agents are intracellular parasites of macrophages of spleen, lymph nodes, bone marrow and liver.

Disease expansion has been attributed to the climatic changes affecting the distribution and habits of the insect vector [5]. Control efforts for VL include the treatment of human cases, the culling of infected dogs and insecticide spraying in residences [6]. Three canine vaccines have been used for dog prophylaxis [7–10], but no human vaccine is yet available. The disease is lethal if not treated after the onset of the symptoms and 10% of human mortality occurs even after treatment [11].

First-line leishmaniasis drugs are Glucantime and Pentostan antimonials [12]. Their disadvantages include high toxicity (vomiting, arthralgia, hepatitis, pancreatitis and cardiac dysrhythmias), high cost [11], resistance issues in India [13,14] and in HIV patients [4, 15, 16] and intravenous administration during hospitalization. The intravenous alternative drug, Amphotericin B, alone or in liposomal formulations [13,14,17], causes fever, nephrotoxicity and hypokalemia [18]. It also requires hospitalization but is not related to major resistance problems. Paromomicin, of comparable efficacy to Amphotericin, induces nephrotoxicity and ototoxicity. Pentamidine therapy in India was halted because of its decreased cure rate and adverse side effects [17, 19]. Recently, oral therapy with Miltefosine showed 94% of success rates in India [20] but only 50% of cure rates in Brazil [21]. The same compounds are used for the therapy of canine leishmaniasis in Europe, with controversial success [22]. The use of combined therapies reduces time and cost of treatment and avoids the selection of resistant parasites [14, 23]. Protozoan parasites lack *de novo* purine synthetic pathways and have developed robust salvage pathways, where no single enzyme is predicted to be essential [24]. The inhibitory potential of iminoribitols substituted with aromatic groups against nucleoside hydrolases (NH) of protozoan parasites was established in the 90s and led us to test them here [25–27]. The nucleoside hydrolase NH36 of *L*. *(L*.*) donovani* is also the main antigen of the Leishmune vaccine used for prevention and therapy of canine visceral leishmaniasis [5, 7, 8].

Immucillin ImmA (IA) and ImmH (IH) are examples of synthetic deazapurine iminosugar-C-nucleoside synthetic transition state analogues [28]. We recently assayed the effects of IA, IH, SMIH and of immucillins DADMe-ImmA (DIA), DADMe-ImmH (DIH), DADMe-ImmG (DIG), SerMe-ImmG (SMIG) and SerMe-ImmA (SMIA) on the *L*. *(L*.*) donovani* recombinant NH36 enzymatic activity [29]. IA and IH inhibited the NH36 enzymatic activity with *Ki* = 0.080 μM for IA and 0.019 μM for IH. Inhibition of the growth of *L*. *(L*.*) infantum chagasi* and *L*. *(L*.*) amazonensis* promastigotes *in vitro* was obtained using nanomolar to micromolar concentrations of IA, IH, DIH, DIG, SMIH and SMIG. As transition state analogues of N-ribosyl transferases they are stable chemical mimics of the enzymatic transition state and bind tighter than the respective substrate molecules. Of these, IA, IH and SMIH at 10 μM concentration inhibited 95% of the intracellular replication of *L*. *(L*.*) infantum chagasi* amastigotes *in vitro* causing no apparent damage to macrophage viability. IA and IH were less toxic and more potent than Glucantime [29].

Our results of the *in vitro* model suggested that IA, IH and SMIH might provide new chemotherapy agents for leishmaniasis [29]. Here we tested their efficacy and toxicity compared to those induced by the standard treatment with Glucantime, on BALB/c mice and CB hamsters infected with *L*. *(L*.*) infantum chagasi*.

## Methods

### Ethics statement

All mouse and hamster experiments were performed following the guidelines of the National Institutes of Health, USA and the protocols were reviewed and approved by the Animal Care and Use Committee of the Instituto de Biofísica Carlos Chagas Fo.-UFRJ (CAUAP-CONCEA, Brazil, IMPPG-016). Animals were maintained in the facilities of Instituto de Microbiologia Paulo de Góes da UFRJ, with controlled temperature, 12h light /dark cycles and given water and feed *ad libitum*. Animals were euthanized with CO2. We made all efforts in order to minimize animal suffering.

### Infection, chemotherapy, clinical and parasitological follow–up

**Fig 1:**
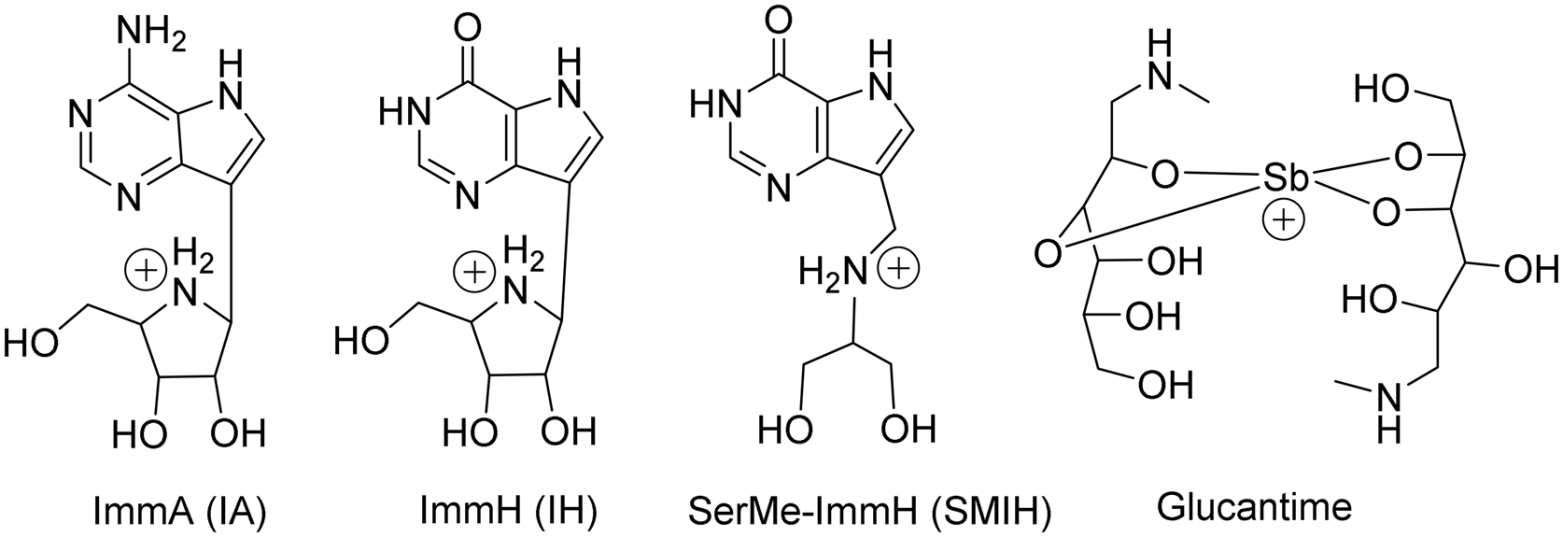
Structures of the compounds used in this study. The structure shown for Glucantime is one of several related antimony chelates found in the drug, which is formed from the reaction between pentavalent antimony and *N*-methyl-D-glucamine.

Female BALB/c mice, 8 week old, were infected through the caudal vein with 10^7^ amastigotes of *L*. *(L*.*) infantum* (strain IOC-L 3324) isolated from infected hamsters spleens. After 15 days of infection, mice (n = 5 per treatment) were injected by the intraperitoneal route (ip) with daily doses for 5 days of 2.5 mg/Kg of IA, IH, SMIH [30, 31] or the control drug Glucantime (Sanofi Aventis, batch 0929280802) (Fig 1). Immucillins were synthesized at the Ferrier Research Institute, Victoria University of Wellington, New Zealand. Uninfected and infected mice treated only with saline were included as controls for the treatment cohorts. On days 1, 15, and 30 after complete treatment [31, 32], the animals were euthanized with CO_2_. Blood samples were collected for sera analysis and weight of spleens, livers and total body were determined. Spleens were also used for determination of cytokine expression. The parasite load was evaluated in Giemsa-stained liver smears and expressed in LDU values (Leishman Donovan units of Stauber = number of amastigotes per 1000 liver cell nuclei/mg of liver weight). The levels of urea, creatinine, glutamate pyruvate transaminase (GPT) and glutamate oxaloacetate transaminase (GPO) were assessed in plasma by standard clinical laboratory procedures using a Wiener lab Metrolab 2300 (Laborlife Análises Clínicas laboratory, Rio de Janeiro).

The efficacy of immucillins was also assayed in the CB golden hamster susceptible model. Females, 8 weeks old, were infected through the intracardiac route with 2 x 10^7^ amastigotes of *L*. *(L*.*) infantum chagasi* (strain IOC-L 3324) isolated from infected hamsters spleens. Thirty days after infection, the animals were treated with 2.5 mg/kg daily doses of Glucantime, IA, IH or SMIH, through the ip route, for five days. Ten days after complete treatment the animals were euthanized, their parasite load was determined in livers and the renal function evaluated in sera samples as described above.

### Effect of Immucillins on cell mediated immunity

Female BALB/c mice, 8 weeks old, were injected intraperitoneally with daily doses 2.5 mg/Kg of IA, IH, SMIH or Glucantime during five days and euthanized on day 1, 15 and 30 after complete treatment. For evaluation of the T cell response, spleens were removed and splenocytes were incubated for 24 h *in vitro* with the lysate of 10^6^ stationary phase promastigotes of *L*. *(L*.*) infantum chagasi* (MHOM/BR/1974/PP75), with 25 μg/mL NH36, at 37°C and 5% CO_2_, during 72 h for the assay of the cytokine expression in the supernatants. Additionally, splenocytes from uninfected BALB/c mice either untreated or previously treated with IA, IH, SMIH or Glucantime were incubated *in vitro* with 10 μg/mL of each respective drug. For flow cytometry analysis (FACS analysis), splenocytes were incubated for 24 h with the antigens or drugs, labeled with anti-CD4FITC (clone GK1.5), anti-CD8FITC (clone 53–6.7) monoclonal antibodies (R&D systems, Inc) or with rat anti-mouse CD19-PerCP-Cy5.5 and 100,000 lymphocyte counts were acquired using a BD FACScalibur apparatus. Data was analyzed using the Flow-Jo program. The secretion of cytokines was evaluated in the supernatants of splenocytes by an ELISA assay.

### ELISA

The NH36 gene of *Leishmania (L*.*) donovani* (EMBL, Genbank and DDJB data bases, access number AY007193) [33] with a His_6_tag at the C-terminal was cloned in *E*. *coli* Bl21DE3. Expression of the recombinant NH36 was obtained by induction with 0.5mM IPTG and overnight incubation at 20°C. Cultures were harvested by centrifugation and the pellets submitted to sonication during 10 minutes with cycles of 30 sec. and intervals of 55 sec. Sonicates were centrifuged and their supernatants separated for purification by column chromatography using Ni-NTA Superflow resin (Qiagen, USA). The column was eluted with a 50mM potassium phosphate, 300 mM NaCl buffer, pH: 8.0, using a 50 to 300mM imidazole gradient [29]. The fraction containing NH36 recombinant antigen was recovered, dyalized and preserved at -80°C. Sera were collected from mice by intracardiac puncture on day 1, 15 and 30 following complete chemotherapy and assayed for the presence of anti-NH36 IgG, IgG1 and IgG2a antibodies. For that purpose the recombinant NH36 antigen (40 μg/mL) was solubilized in 0.1M sodium carbonate buffer (8.4 g of NaHCO_3_, 3.56 g of Na_2_CO_3_ per L; pH 9.6), and used to coat flat-bottom 96-well plates. Antibodies were detected by goat anti-mice IgG (Sigma), anti-IgG1 and anti-IgG2a peroxidase-conjugates (Southern Biotechnology Associates, Birmingham, AL, EEUU) in a 1:1000 dilution [33]. The absorbance values at 492 ηm were compared using a 1:100 dilution of the serum samples. Results are expressed as mean values of triplicates. We used the mean average + 2 SD of serum from normal uninfected controls in order to calculate the cut-off value according to the Youden test [34].

### Cytokine expression

Splenocytes were obtained from euthanized mice on days 1, 15 and 30 after complete chemotherapy, as described previously [33] and platted (10^6^ cells/ well) in serum supplemented RPMI medium with no additions, together with the lysate of 10^6^ stationary phase promastigotes of *L*. *(L*.*) infantum chagasi* (MHOM/BR/1974/PP75), or with 25 μg/mL NH36. Cells were incubated for 72 h with 5% CO_2_ at 37°C, according to previous standardization experiments. The secretion of IFN-γ, TNF-α, IL-10 and IL-4 were analyzed in supernatants using e-Bioscience (San Diego, CA, USA) ELISA-assay kit following the manufacturer instructions. Recombinant IFN-γ (2000 pg/mL–15.6 pg/mL), TNF-α (2000 pg/mL-15.6 pg/mL), IL-10 (4000 pg/mL-15.6 pg/mL) and IL-4 (500 pg/mL-4 pg/ml) were used as standards. Reactions were developed with 100 μL/well TMB (Zymed) and absorbances were recorded at 405 nm by an ELISA BioRad Benchmark.

### Statistical analysis

We used the non-parametrical Kruskall Wallis and Mann Whitney tests (GraphPad Prism6 program) for comparison of means. Correlation coefficient analyses were determined with a Pearson bivariate, two-tailed test of significance (GraphPad Prism6 program).

## Results

### Efficacy of treatments in mice

**Fig 2:**
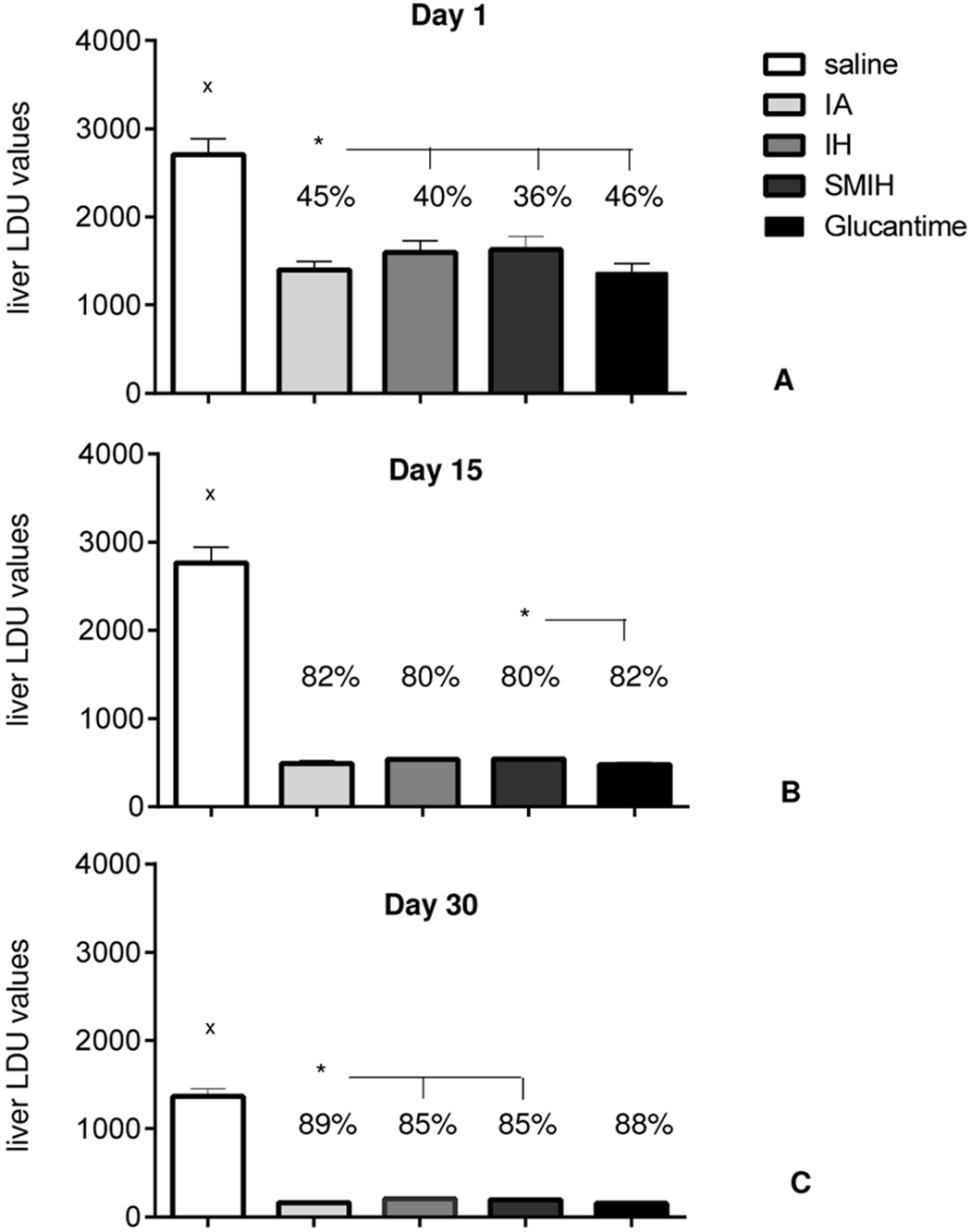
*L*. *(L*.*) infantum chagasi* parasite load in liver determined in Leishman Donovan units of Stauber on days 1 (A), 15 (B) and 30 (C) after complete treatment. Small x indicates significant differences from all treatments; asterisks and horizontal lines indicate significant differences between groups. Values are mean + SE of 10 animals (two independent experiments with n = 5 per group).

The therapeutic efficacy of test drugs against VL was evaluated by *L*. *(L*.*) infantum chagasi* parasite burden in isolated mouse livers (Fig 2). All treatments reduced the liver parasite load, if compared to untreated controls (*P* < 0.0001). Drug efficacy was evident even on day 1 after the end of treatment (*P* < 0.0001). IA and Glucantime reduced the parasite load by 45% and 46%, respectively, at day 1 (Fig 2A). The parasite load also decreased at longer times following therapy (Fig 2B and 2C). IA exhibited the strongest therapeutic effect of the immucillins (89%) at day 30, followed by IH (85%) and SMIH (85%) (Fig 2C). Furthermore, a 100% survival was observed at the end of the experiment in infected mice treated with IA, IH, SMIH and Glucantime, and all that showed reduced parasite burden. Untreated infected mice had higher parasite burdens and lower survival rates (90%).

**Fig 3:**
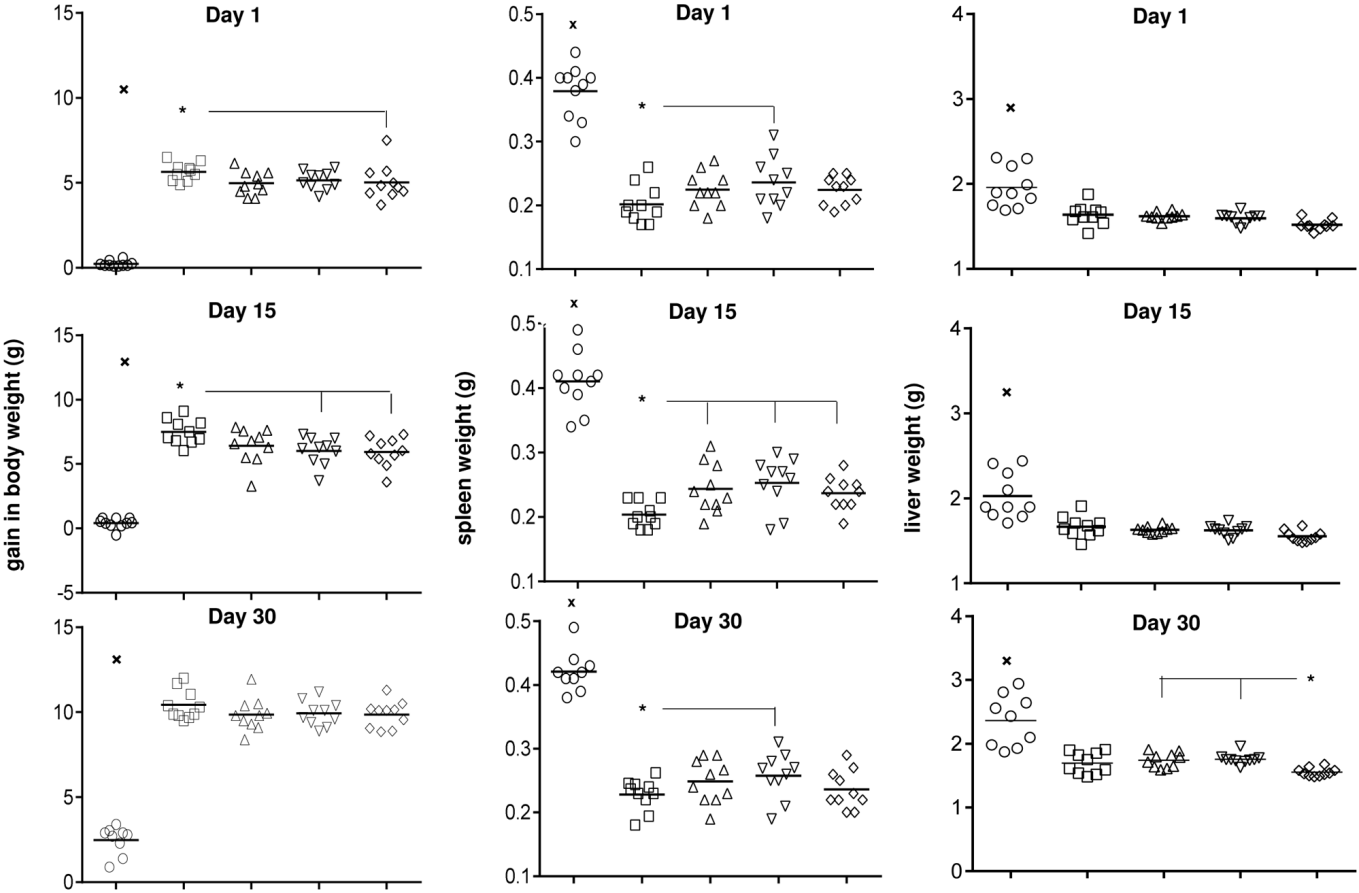
Increase in body, spleen and liver weight as a function of time following the treatment. Small x indicates significant differences from all treatments; asterisks and horizontal lines indicate significant differences between groups. Values are mean + SE of 10 animals (two independent experiments with n = 5 per group).

Infected mice resist weight gain if untreated, and body weight gain was significantly increased (*P* < 0.0001) in all chemotherapy groups (Fig 3). Gain of body weight in group treated with IA was significantly higher compared to animal group treated with Glucantime on day 1 (*P* < 0.05) and higher than that observed in groups treated with SMIH (*P* < 0.05) and Glucantime (*P* < 0.02) on day 15 after the end of treatment. As expected for VL, the spleen weight of infected saline treated animals, showed a significant increase from day 1 (mean = 0.38 mg) to day 30 (0.42 mg; p = 0.0289) (Fig 3). All chemotherapy treatments reduced the weights of spleens (~ 58%). IA was the most effective and induced stronger curative effects (*P* < 0.040) than SMIH on day 1 and day 30, and than IH, SMIH and Glucantime (*P* < 0.013) on day 15 (Fig 3). Also, as expected for VL, the liver weight of saline controls exhibited significant increases from day 1 (mean = 1.96 mg; p = 0.0220) and day 15 (2.03 mg p = 0.0367) to day 30 (2.36 mg) (Fig 3). The liver weight was reduced by all treatments. On day 30 following treatment, Glucantime was slightly more potent than SMIH and IH but not different from IA in reducing liver weight (Fig 3).

The clinical features correlated with the parasite burden for VL. Liver LDU values were correlated to the spleen (*P* <0.0001; R = 0.7905, R^2^ = 0.6249) and liver (*P* <0.0001; R = 0.6652; R^2^ = 0.4424) weights. Both are also correlated to each other (*P* <0.0001; R = 0.5243; R^2^ = 0.2749). Liver parasite burden (LDU values) (*P* < 0.0001; R = -0.8481; R^2^ = 0.7193), spleen weights (*P* <0.0001; R = -0.6512; R^2^ = 0.4240) and liver weights (*P* <0.0001; R = -0.4400; R^2^ = 0.1936), were negatively correlated to gain in body weight.

### Serum antibody response

**Fig 4:**
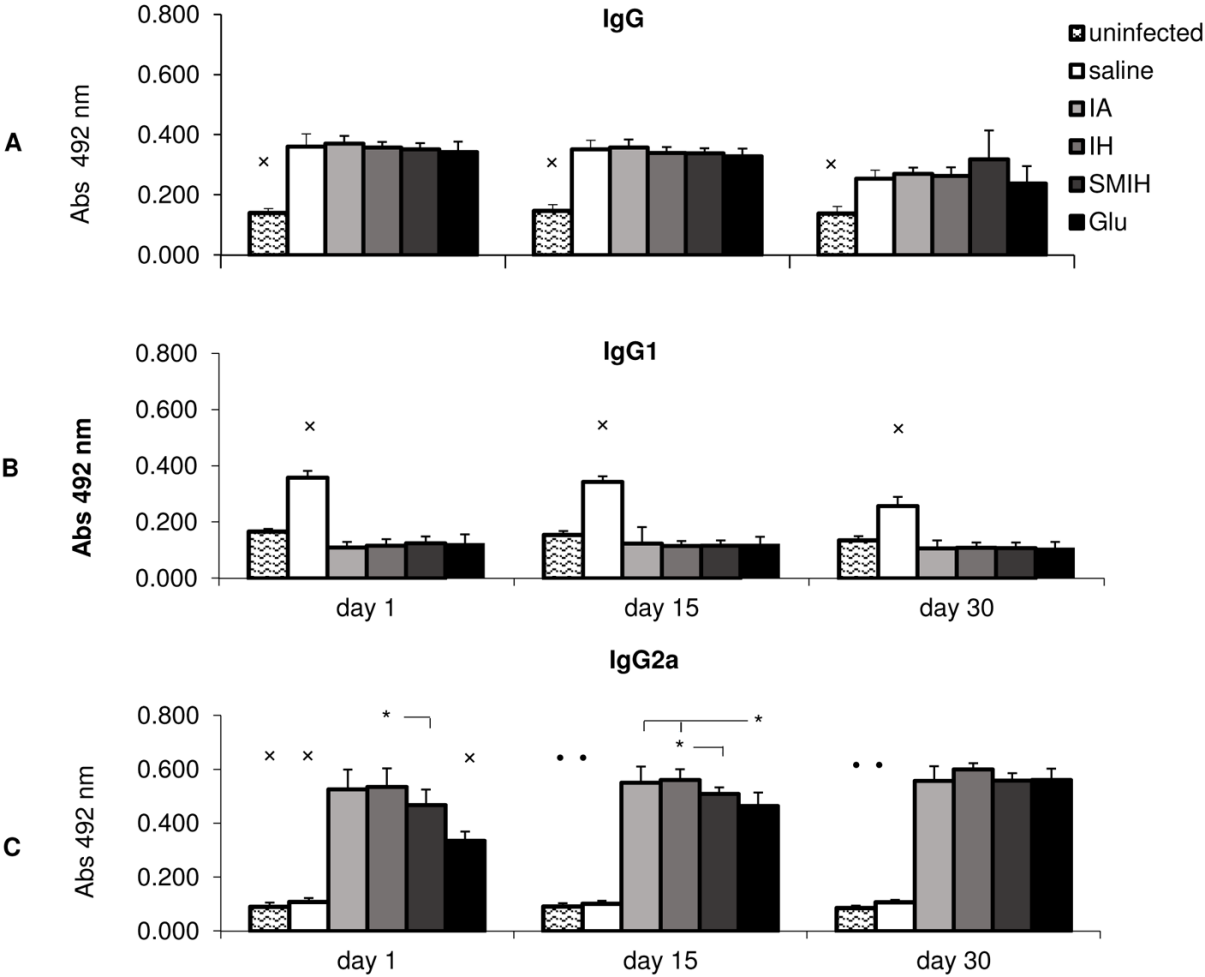
Humoral response in mice infected with *L*. *(L*.*) infantum chagasi* and treated with immucillins and Glucantime, at timed intervals following 5 days of drug therapy. The anti-NH36 specific IgG, (A), IgG1 (B) and IgG2a (C) antibodies were assayed by ELISA in sera of infected and non-infected mice, at 1, 15 and 30 days after complete treatment with IA, IH, SMIH or Glucantime (2.5mg/kg/day during 5 days). Small x represents significant differences from all other treatments; filled circles: significantly different from IA, IH, SMIH and Glucantime treatments; asterisks and horizontal lines indicate significant differences between groups. Values are mean + SE of 10 animals (two independent experiments with n = 5 per group).

A significant increase in anti-NH36 IgG antibodies was observed in all infected animals (treated or not) including day 30 following treatment (*P* < 0.05) (Fig 4A). Using the mean average of IgG absorbances + 2 SD of serum of normal uninfected mice we obtained the cut-off values: 0.168 for day 1, 0.147 for day 15 and 0.184 for day 30 after infection, respectively. All these cut-off values obtained the Youden index = 1 a value which indicates the absence of false positive and false negative results. The set of antibody means of normal uninfected and of infected mice differ by 15 (day 1), 10 (day15) and 5 (day 30) standard deviations, respectively, indicating the accuracy of the test. The anti-NH36 IgG1 antibody absorbencies were enhanced only in the infected untreated controls (*P* < 0.0001) where the increases were 69% and 64% higher than in IA treated mice (days 1 and 15, respectively). There was a slight decrease to 57% at day 30 following chemotherapy (Fig 4B). IgG1 antibody responses suggested that the efficacy of immucillins in control of infection is compatible with that of Glucantime. However, the results of the IgG2a subtype indicated that a stronger antibody response is induced by immucillins (Fig 4C). Immucillins and Glucantime generated high anti-NH36 IgG2a responses (Fig 4C) if compared to untreated infected controls, which remained at basal levels (*P* <0.001). IgG2a increases in the IA, IH and SMIH groups (79–77%) started from 1 day following chemotherapy, with no decline until day 30, suggesting a rapid onset of infection control. Glucantime treatment, resulted in a slower IgG2a response (67% at day 1) (*P* < 0.001), and reached maximal values only at day 30. IA and IH were more potent than Glucantime and IH induced an IgG2a stronger response than SMIH (day 1 and 15) (Fig 4C).

### Cytokine response

**Fig 5:**
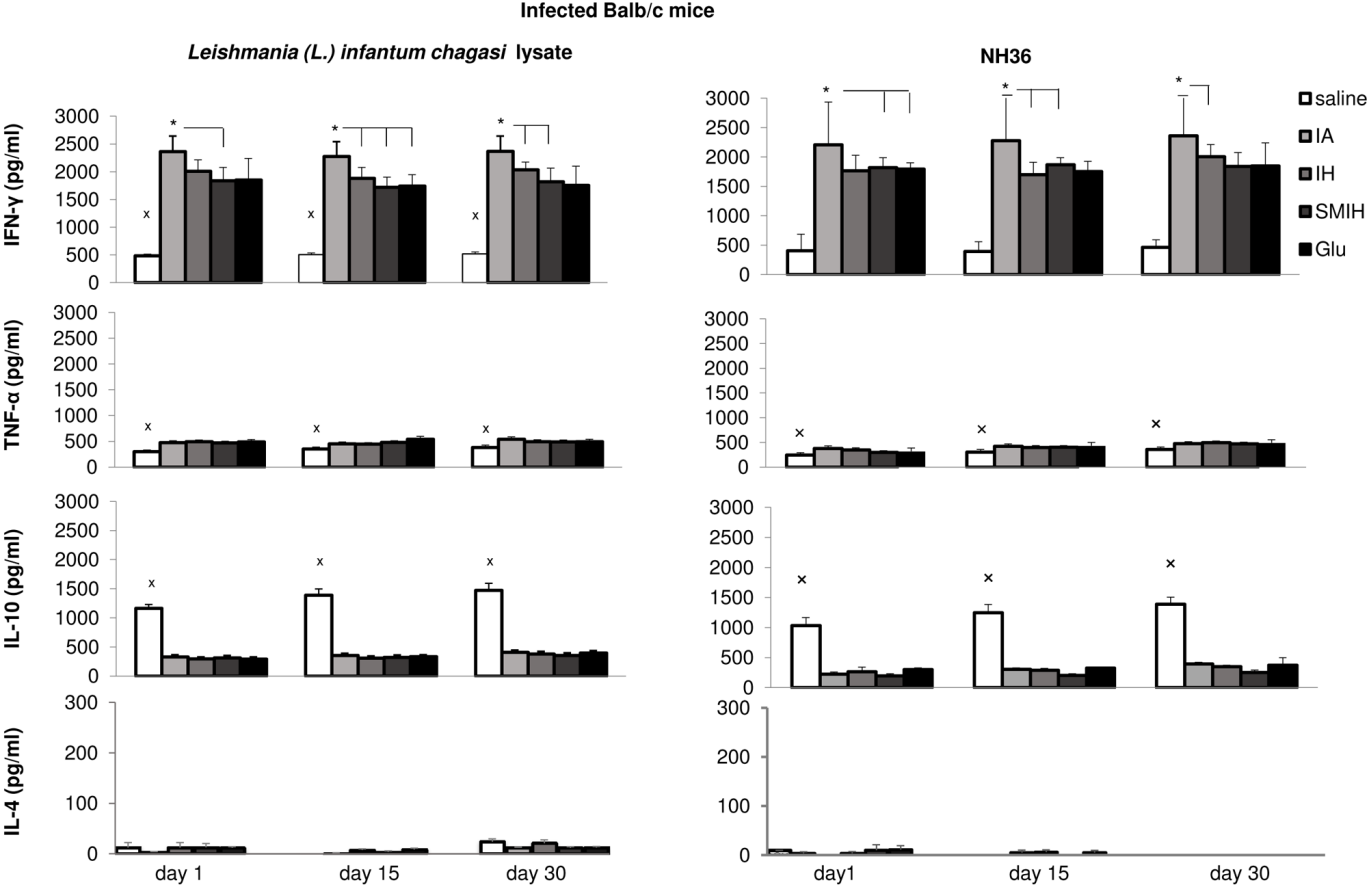
*Leishmania* antigen-specific cytokine secretion response assayed by ELISA in the supernatants of splenocytes of infected and treated mice incubated *in vitro* with *L*. *(L*.*) infantum chagasi* lysate or with NH36. Small x indicates significant differences from all treatments; asterisks and horizontal lines indicate significant differences between groups. Values are mean + SE of 10 animals (two independent experiments with n = 5 per group).

IFN-γ levels were significantly enhanced in all groups receiving chemotherapy, when compared to untreated controls (Fig 5A and 5E). IFN-γ was most increased following treatment with IA (79–78% against the *L*. *(L*.*) infantum chagasi* lysate and 82–80% against NH36). IA was more active than other immucillins or than other immucillins and Glucantime in response to the *Leishmania* antigen (Fig 5A). IA and IH were similar in a response to the NH36 antigen (Fig 5E). Secretion of TNF-α in response to both antigens was mildly enhanced above controls in all groups that received chemotherapy (Fig 5B and 5F). IL-10 secretion was significantly enhanced (75%; *P* < 0.001) in infected untreated mice in response to both antigens, at all times assayed (Fig 5C and 5G). Secretion of IL-4, was low, 100 times smaller than IFN-Ɣ, either after stimulation with lysate (it was < 25 pg/ml) (Fig 5D) or NH36 (it was < 11 pg/ml) (Fig 5H), with no differences between test times or treatments.

Regarding the correlation analysis, the level of anti-NH36 IgG1 immunoglobulin is considered a good indicator of the disease. It was correlated closely with liver LDU values (*P* <0.0001; R = 0.7267; R^2^ = 0.5281), while IgG2a, a correlate of therapeutic efficacy, was negatively correlated to LDU values (*P*<0.0001; R = -0.7335; R^2^ = 0.5380).

Furthermore, IL-10, a hallmark of the disease, correlated with LDU values (p<0.0001; R = 0.6447; R^2^ = 0.4157 for *Leishmania* lysate and p<0,0001; R = 0.5306; R^2^ = 0.2816, for NH36), while IFN-γ, a good marker of therapeutic efficacy, was negatively correlated to LDU values (p <0.0001; R = -0.4457; R^2^ = 0.1986 for *Leishmania* antigen and p<0.0001, R = -0,5121; R^2^ = 0,2623 for NH36). A similar negative correlation with LDU values was observed for TNF-α secretion (p <0.0019; R = -0.2523; R^2^ = 0.0636 for *Leishmania* antigen and p<0.0001, R = -0.4261; R^2^ = 0.1815 for NH36).

### Cellular immunity induced by immucillins

**Fig 6:**
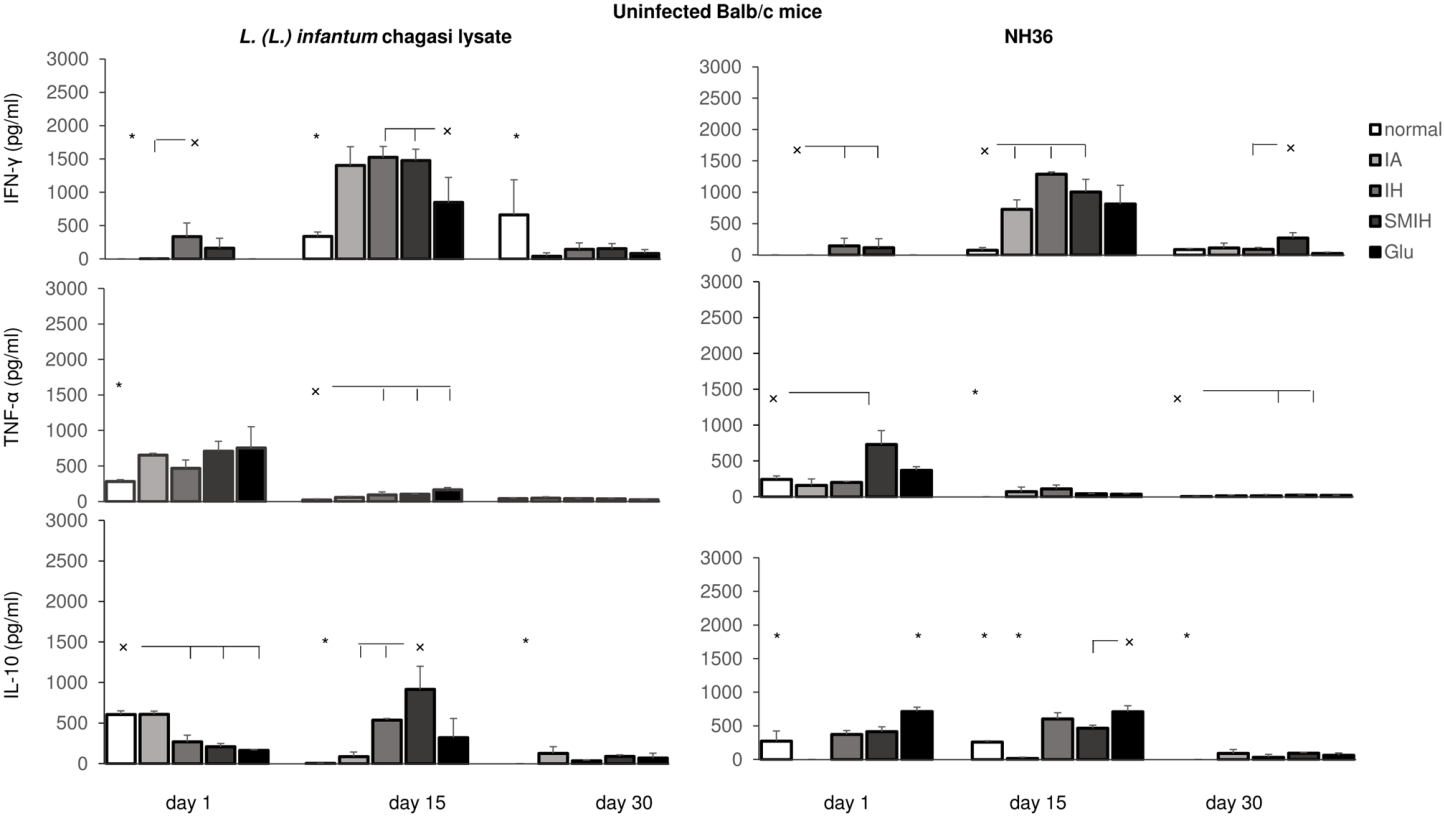
*Leishmania* antigen-specific cytokine secretion response assayed by ELISA in the supernatants of splenocytes of uninfected mice treated with IA, IH, SMIH or Glucantime and incubated *in vitro* with *L*. *(L*.*) infantum chagasi* lysate or with NH36. Asterisks indicate significant differences from all treatments. Small x and horizontal lines indicate significant differences between groups. Values are mean + SD of 3 animals per treatment.

The remarkable change in cellular and humoral immune responses observed after immucillin treatment (Figs 4 and 5) could be due to the decrease in parasite load but also to the direct action of immucillins on the immune system. To explore this hypothesis we treated normal uninfected mice with each immucillin or with Glucantime, and incubated their splenocytes *in vitro* in the presence of lysate of *L*. *(L*.*) infantum* or NH36 (Fig 6). The secretion of IFN-γ in response to lysate or NH36 was enhanced only on day 15, in mice treated with all drugs. Immucillins promoted a higher IFN-γ than Glucantime (Fig 6). The response of uninfected animals (Fig 6) however, was 35% lower than in uninfected animals (Figs 5 and 6) and transient. Different from infected animals (Fig 5), a slight enhancement of TNF-α secretion was observed in uninfected mice, only on day 1, which did not last (Fig 6). This was induced in response to the lysate by all drug treatments, and to NH36, only in SMIH treated mice (Fig 6). As expected, the IL-10 secretion of normal mice was reduced by 47% and 73%, in response to lysate and NH36, respectively, when compared to those of infected saline-treated controls (Fig 5). The IL-10 response was mainly increased in SMIH treated mice, to lysate, and in Glucantime treated animals, in response to NH36 (Fig 6). We conclude that the overall cytokine response of immucillin and Glucantime treated uninfected animals suggests that immucillins might also have a moderate but direct effect on T cells which contributes to the cure of VL.

**Fig 7:**
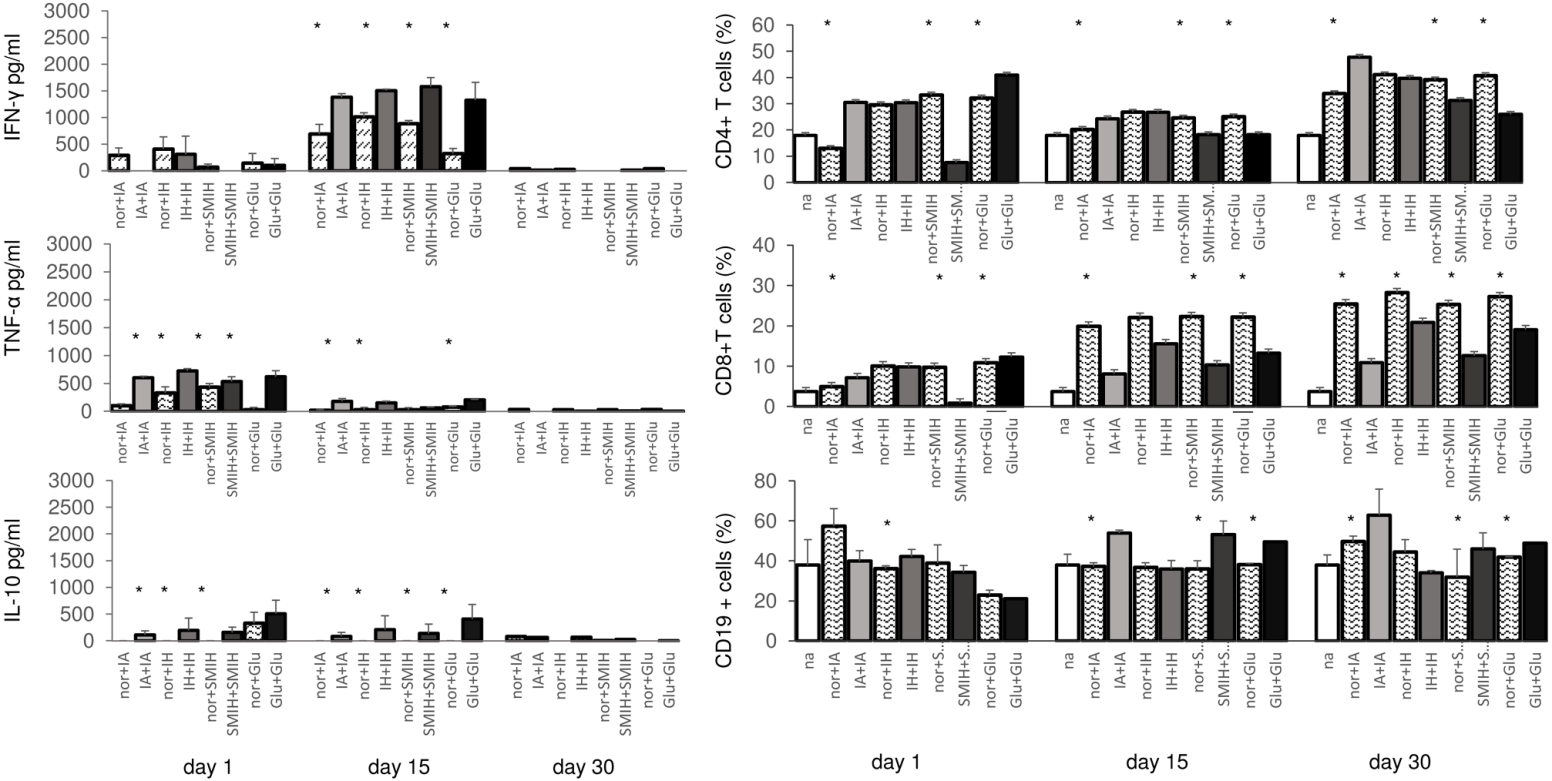
Immucillin directly stimulate cytokine expression of T cells. Splenocytes of normal untreated and uninfected mice (nor+IA, nor+IH, nor+SMIH, nor+Glu) and of mice previously treated with IA (IA+IA), IH (IH+IH), SMIH (SMIH+SMIH) or Glucantime (Glu+Glu) were incubated *in vitro* with 10 μg/ml of the respective drug. Control cells were incubated with no addition (na). The induced cytokine expression in supernatants was measured by an ELISA assay and the frequencies of CD4+, CD8+ and CD19+ lymphocytes were assessed by flow cytometry. Values are mean + SD of 3–5 animals per group. Asterisks indicate significant differences between splenocytes of normal untreated and of previously treated mice. Small x indicate significant differences from all groups.

To confirm this possibility we incubated splenocytes of normal untreated mice and mice previously treated with immucillins or Glucantime, with each respective drug *in vitro* (Fig 7). All immucillins and Glucantime increased the secretion of IFN-γ by ~50% (day 15) in the supernatant of drug treated mice. This level reaches 35% of the secretion of IFN-γ by splenocytes of infected animals (Fig 5). A similar pattern, but to a lower extent, was observed for the secretion of TNF-α, at day 1, and for the secretion of IL-10, with the exception of SMIH, at day 15 (Fig 6). Noteworthy, IL-10 levels in Glucantime treated mice (509 pg/ml) were higher than in IA treated mice (113 pg/ml; p<0.05). Levels of IFN-γ were 3 times higher (~1500 pg/ml) than those of TNF-α (~500 pg/ml) (Fig 6). Although the levels of IFN-γ in mice treated after infection (Fig 5) were 35% higher (2,200 to 2,370 pg/ml) than in uninfected treated mice (Fig 7), mainly in the case of IA, we demonstrated that a robust cytokine response is induced in lymphocytes by immucillin treatments.

Furthermore, the effect of immucillins is evident in the increase of lymphocyte populations in treated mouse spleens (Fig 7). IA induced the most pronounced enhancement of CD4+ T cells frequencies, showing 57%, 21% and 29% higher proportions than the levels of the previously untreated controls, on days 1, 15 and 30, respectively (Fig 7). IH also enhanced CD4+ T cell counts however, on both, treated and untreated mice, while Glucantime, promoted a high CD4+ T cell increase (p<0.05) only on day 1. SMIH treated mice showed lower CD4+ T cell frequencies (Fig 7). Our results suggest that the decrease in parasite load promoted by immucillins is due their inhibitory effect on parasite replication and to the induction of a TH1 immunogenic response.

On day 1 after treatment, the CD8+ T cell frequencies were increased by IA and Glucantime. IH, as detected for CD4+ T cell frequencies, promoted unspecifically the increase of CD8+T cells on both, previously treated and control mice, while SMIH diminished CD8+T cells proportions in treated mice. Furthermore, the CD8+ T cell frequencies were diminished by all drugs at longer times after therapy (Fig 7).

Regarding the CD19 B cell response, frequencies were ~30% enhanced by previous treatment with IA, SMIH and Glucantime and sustained by therapy with IH (Fig 7).

### *In vivo* toxicity in mice

**Fig 8:**
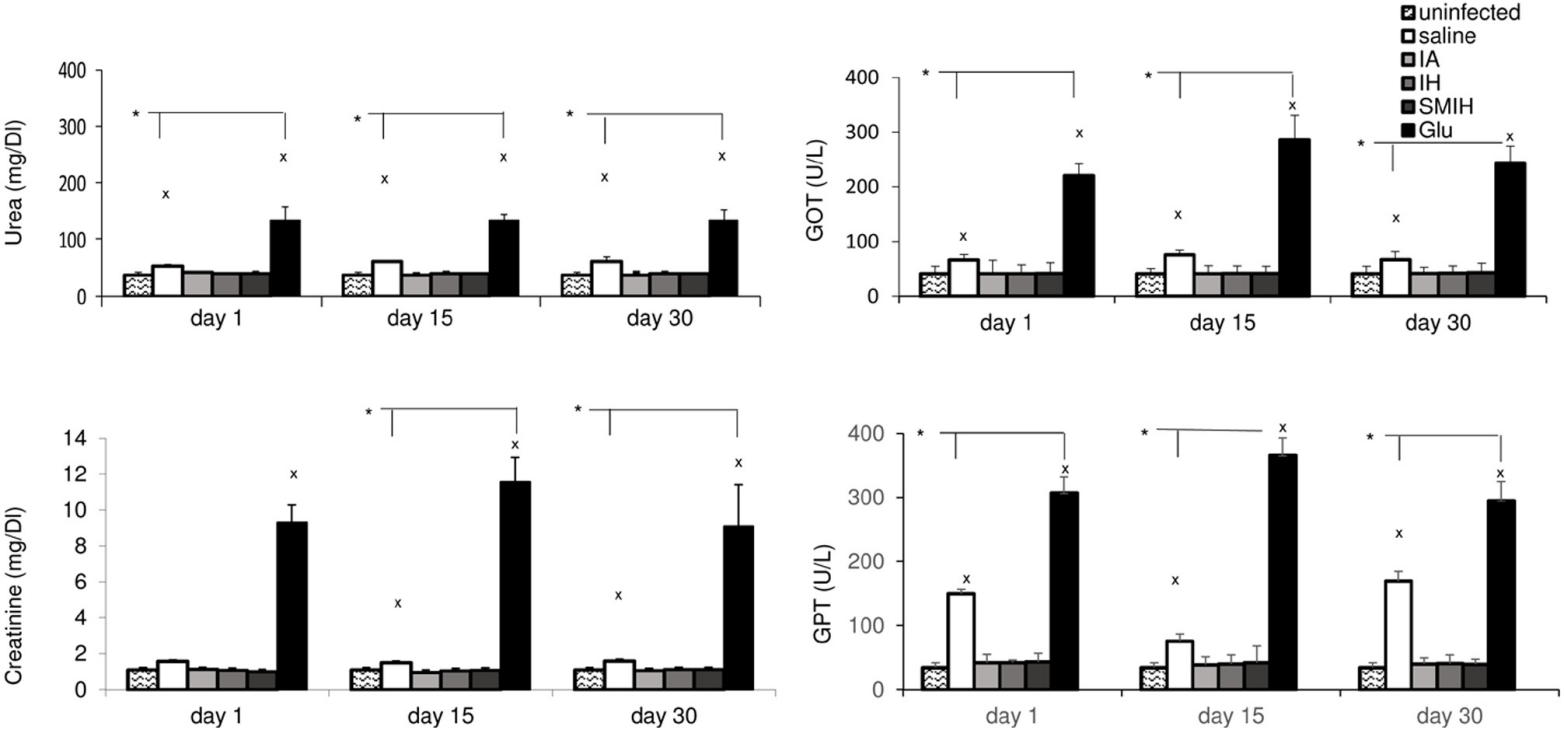
Renal and liver function markers in plasma of mice as a function of time following experimental therapy. Small x indicates a group significantly different from all treatments; asterisks and horizontal lines indicate significant differences between groups. Values are mean + SE of 10 animals (two independent experiments with n = 5 per group).

Immucillin treatments caused no adverse changes in renal (urea and creatinine) or hepatic (GOT and GTP) function (Fig 8). Pronounced toxicity was a consequence of the treatment with Glucantime. Increases in urea (4-fold), creatinine (9-fold), GOT (6-fold) and GPT (9-fold) serum levels resulted from Glucantime therapy (Fig 8). Infected, untreated mouse controls showed lower responses in these parameters when compared to normal untreated mice (*P* < 0.0001) (Fig 8). Although the therapeutic efficacy of immucillins and Glucantime were compatible, treatment with immucillins prevented toxicity.

### Efficacy and *in vivo* toxicity in hamsters

**Fig 9:**
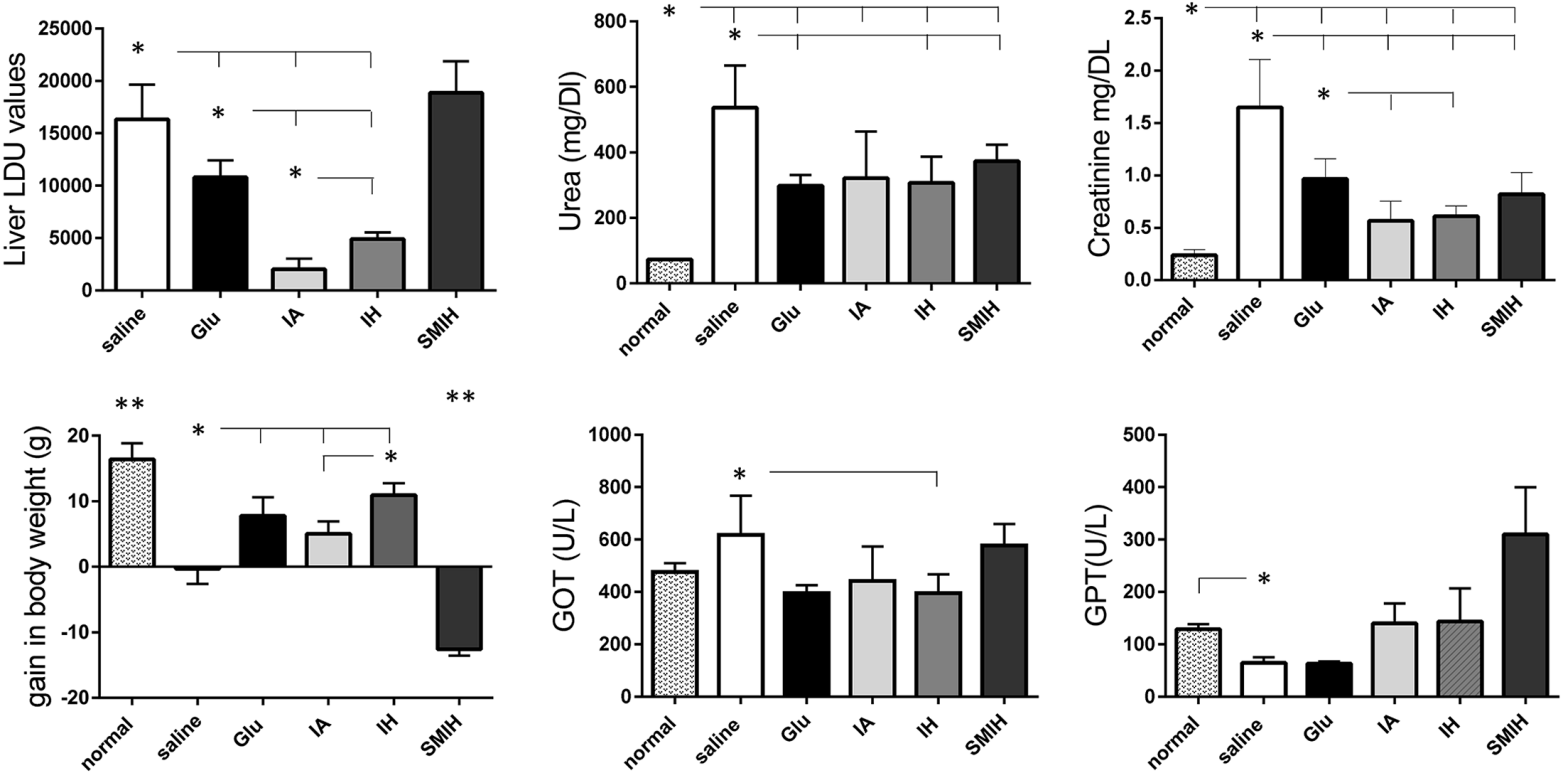
Liver parasite load and renal function markers in sera after chemotherapy of hamsters. Asterisks and horizontal lines indicate significant differences between groups. Two asterisks indicate significant differences from all other group. Values are mean + SE of 10 CB hamsters animals (two independent experiments with n = 5 per group).

The efficacy of immucillins was further assessed in the susceptible hamster model (Fig 9). Hamsters developed a greater liver parasite load (16,334 LDU) (Fig 9) than BALB/c mice (2,765 LDU) (Fig 2), but gave similar responses to immucillins (Figs 2 and 9). Parasite load reductions of 86% and 70% were induced by IA (2,038 LDU) and IH (4,905 LDU) treatments, respectively (p < 0.001). Treatment with SMIH gave no cure (Fig 9). Accordingly, an increase in body weight was promoted by IA, IH and Glucantime, but not by SMIH (Fig 8). *Leishmania* infection promoted a high increase of blood urea levels, that were reduced by ~50% by Glucantime, IH and SMIH. All drugs, promoted a decreased of creatinine to normal levels (Fig 8) which was more pronounced after IA (66%) or IH (63%) than after Glucantime (41%) treatment. GOT levels increased after infection and IH, but not other immucillins, reduced them by ~ 30%. GPT was unchanged.

We conclude that, in mice, immucillins IA, IH and SMIH show higher potency and earlier onset of generation of IgG2a antibodies than the standard drug Glucantime. IA induced higher secretion of IFN-γ, more gain in body weight and a better reduction of spenomegaly than Glucantime, while causing similar levels of liver parasite burden and much lower toxicity. The direct action of IA, IH and SMIH on mice lymphocyte responses partially contribute to the success of chemotherapy of VL. IA induces a TH1 response, followed by IH. Although all immucillins were strongly therapeutic in mice, a superiority of IA was observed in most of the variables. This probably explains why IA is the most effective against VL in hamsters where SMIH show no therapeutic effect.

## Discussion

Transition-state theory has led to the design of immucillins that inhibit the enzymatic activity of nucleoside hydrolases of parasitic protozoa [28, 35]. IA inhibits the NH of *Chrithidia fasciculata*, *Trypanosoma brucei brucei*, [24], *L*. *(L*.*) major* [35] and *L*. *(L*.*) donovani* [29], the purine nucleoside phosphorylase (PNP) of *Trichomonas vaginalis* [36] and the viral RNA polymerases of Marburg and Ebola filovirus [37]. Human PNP of erythrocytes and lymphocytes and human 5, methylthioadenosine phosphorylase have also been identified as targets of IA in humans [38]. Furthermore, IA also inhibits the replication of *L (L*.*) infantum chagasi*, *L*. *(L*.*) amazonensis* [29], and showed broad-spectrum antiviral activity against numerous viruses, including togavirus, bunyaviruses, arenaviruses, paramyxoviruses, coronaviruses orthomyxovirus, Picornavirus and Flavivirus with untested targets [37]. IA has been shown to be safe in primates, and is now in phase 1 clinical trials for humans under the name BCX4430 [37].

Another immucillin, IH inhibits the PNP of *Toxoplasma gondii* [39], the NH of *Leishmania (L*.*) donovani* [29] and human PNP [40–42]. Additionally, IH reduces the replication of *L (L*.*) infantum chagasi*, *L (L*.*) amazonensis* with as yet undefined targets [29]. Immucillin IH has been in more than a dozen clinical trials (under the name Forodesine) for humans, and is considered to have acceptable side effects [43].

Finally, SerMe-ImmH (SMIH) acts on the PNPs of mice erythrocytes [28] and of human T cells [40], and inhibits the *in vitro* replication *L (L*.*) infantum chagasi* and *L*. *(L*.*) amazonensis* [29] with as yet unknown targets. SMIH however, has not yet been tested for toxicity in animals or man.

A common mechanism of action is however, not established for each compound (IA, IH and SMIH) by the present studies. The possibilities include action at NH for IA and IH that are powerful NH inhibitors [29]; nucleic acid polymerases in the case of IA, where such is known to act in the case of viral RNA polymerases [37]; other enzymes of nucleoside or nucleotide metabolism [38]; and finally, the possibility of acting at other sites such as transport processes. The probable role of NH as one of the Leishmanial targets of immucilin treatment is suggested by the nanomolar inhibition of the *in vitro* activity of the nucleoside hydrolases of *L*. *(L*.*) major* and *L*. *(L*.*) donovani* [29, 35] by IA and IH. IA, IH and SMIH also showed anti-parasite activity against *L*. *(L*.*) infantum chagasi* and *L*. *(L*.*) amazonensis* multiplication *in vitro* with no damage to macrophage viability [29].

In this study we showed that IA, IH and SMIH immucillins are alternative chemotherapeutic agents against VL of mice that show promising efficacy regardless of the mechanism. IA showed the highest curative effect in mice and also promoted cure of VL in hamsters. The enhancement of anti-NH36 IgG antibodies in all infected groups, confirmed that NH36 is an important marker of VL infection, not only in dogs [44] but also, in BALB/c mice. Of note, the anti-NH36 IgG1 antibodies increased only in the infected untreated controls and were reduced by day 30, when the spontaneous control of infection occurs [45]. In agreement with our results with immucillins, much higher levels of IgG1 were found in infected, than in cisplatin treated BALB/c mice [30, 31] or uninfected controls [30] soon after complete chemotherapy. The lower levels of IgG1 and higher titers of IgG2a anti-NH36 antibodies, which correlate to the decreased parasite burden, suggested a pronounced switch to the TH1 response, promoted by the treatments with IA, IH followed by SMIH or Glucantime. Glucantime induction of the IgG2a humoral response was however slower than that of immucillins. Of note, the enhancement of the IgG2a response by immucillins was 80%, by Miltefosin, 75% [46] and by cisplatin, only 50–55% [30, 31].

Supporting the hypothesis of the switch to a TH1 response, the treatment with IA, IH and SMIH immucillins causes IFN-γ to be the predominant secreted cytokine, an effect also observed after treatment with cisplatin [30, 31], and low but detectable levels of TNF-α, which are correlated with resistance to infection and parasite death [47]. Treatment with immucillins or 1,3,4-tiadiazolium-2 aminide compounds [48] induced also higher IFN-γ responses than Glucantime. IL-10 is the hallmark of pathology in VL, and was 72% reduced, 1 day after complete treatment with immucillins, and was 50% and 75% reduced at the same time following treatment with cisplatin [30, 31] or 1,3,4-tiadiazolium-2 aminide [48], respectively. As previously described [47, 49–52] the TH2 response in VL is best represented by IL-10 but not by IL-4. Most reports in literature use promastigote lysate antigen for stimulation of cytokine expression. We showed that NH36 is equally potent reinforcing its relevance as a *Leishmania* infection marker [44, 53, 54]. In our investigation, the remarkable change observed in the immune response could be due to the decreasing in parasite load or to direct immunologic changes induced by immucillins. Such changes have been shown with antimony [55]. Although IFN-γ secretion was high after immucillin treatment in infected animals, 35% lower IFN-γ secretion was also observed in uninfected animals treated with immucillins, either in response to leishmanial antigens or to immucillins. A lower but significant secretion of TNF-α and decreased response of IL-10 were also observed. These results suggest that immucillins are capable of inducing a TH1 response through their direct effect on T cells. This effect may contribute to the successful chemotherapy of VL.

Sodium antimony gluconate (SAG) has been shown to activate both the innate and adaptive immune system by indirectly activating pathways for ROS and NO generation [55], imparting in this way resistance to leishmania infection and reinfection. SAG induces proliferation of T cells but not of B cells and upregulates the IFN-γ receptors [55]. We showed that pre-treatment with IA, IH and SMIH induced significantly high secretion of IFN-γ, followed by TNF-α with a low secretion of IL-10. Enhancements in proportions of CD4^+^-T and CD19^+^ B cells at all times and a transient increase in CD8^+^ T cell frequencies were also promoted by previous treatment only with IA. The increase of CD4+ T cell frequencies by immucillins is noteworthy since resistance to *Leishmania (L*.*) infantum* infection has been shown to be related to the function and frequencies of CD4+ T cells [56–59]. A lower and transient lymphoproliferative effect was determined by Glucantime in our model. IH showed equal stimulation of CD4+ T, CD8^+^T and CD19^+^ B cell expansions in previously treated and untreated mice. Differently, SMIH decreased the CD4+ and CD8+ T cell frequencies but enhanced the proportions of CD19+ B cells. These preliminary results might explain the direct induction of a TH1 response and the strong *Leishmania* antibody response promoted by IA and IH, which contributed to their success in cure of mice VL. Despite the induction of an IFN-γ, TNF-α, IL-10 and antibody response similar to IA and IH, SMIH did not stimulate T cell proliferation and apparently acts via its potent leishmanicidal toxicity [29]. SMIH impaired stimulation on CD4+ T cell proliferation might also explain its lack of efficacy in hamsters, a more susceptible host. We conclude that, as suggested for Glucantime and Pentamidine, the efficacy of IA and IH immucillins is also partially T-cell dependent [60].

Immucillins are potent inhibitors of the PNP activities [37, 61]. IA did not induced mutagenicity or chromosomal aberrations in human lymphocytes and was metabolically stable for mouse, rat, guinea pig and cynomolgus macaque showing rapid clearance from plasma [37]. SMIH has been shown to be orally available in mice inhibiting blood PNP for long periods [28]. IH (forodesine) is also effective as a PNP inhibitor against leukemia cells [61]. In spite of the theoretical concern regarding the association of PNP inhibition and immunodeficiency, we showed that in normal healthy mice, IH stimulates the proliferation of CD4+ and CD8+-T and CD19+-B cells after direct contact or systemic previous treatment.

The correlation of clinical and parasitological variables reinforces the efficacy of the immucillins. While infected untreated mice show small weight gains and increased spleens and livers, therapy with IA led to the highest body weight gain and the lowest spleen weight observed in all therapies, including Glucantime. As described for cisplatin [32], animals treated with the other immucillins and Glucantime also exhibited favorable liver and spleen weight profiles. Most importantly, infected controls developed ~3000 LDU units in livers on day 21 after infection, similar to mice infected with *L*. *(L*.*) infantum chagasi* [33] or *L*. *(L*.*) donovani* [30–32, 46]. Treatment with IA, IH, SMIH or Glucantime caused a decrease in parasite burden starting from day 1 after complete chemotherapy and removing 85–89% of the amastigotes by day 30. IA showed the strongest efficacy (89%) while cisplatin caused reductions of 50% [32], 75% [30] or 80% [31] and Miltefosin [46], of 50%. The early control of infection, may reflect both the direct anti-*Leishmania* therapeutic efficacy of immucillins and their stimulatory effect on the TH1 response. Our clinical, immunological and parasitological results support efficacy of immucillins in therapy of VL with a preference of IA.

In spite of the similar mice parasite load obtained after infection with *L*. *(L*.*) infantum chagasi* [33] or *L*. *(L*.*) donovani* [30–32, 46], and the inhibitory effect of IA and IH on the enzymatic activity of *L*. *(L*.*) donovani* recombinant NH36, and on the *in vitro* growth of *L*.*(L*.*) infantum chagasi* and *L*.*(L*.*) amazonensis* [29], the present study was focused only on the therapy of *L*. *(L*.*) infantum chagasi* infection *in vivo*. Therefore, further studies are necessary in order to assess the cross-species therapeutic potential of immucillins in leishmaniasis caused by other species given the fact that the drug tests were carried out in rodent models far from the natural human or animal reservoir hosts.

New drugs against VL require both efficacy and low toxicity [13, 23]. Glucantime standard dose for human therapy of VL is 20 mg/kg/day for 20 consecutive days. In the BALB/c model the drug was assayed at 20 mg/kg [62] starting from day 5 after infection, for 30 consecutive days or, alternatively, at 10, 25 or 50mg/kg/day [63–65]. Immucillins, are more effective and safe at lower concentrations and in shorter protocols.

Therapy with Glucantime caused an 88% reduction in parasite load but increased levels of GOT and GTP. Elevated hepatic transaminases have also been reported among the most severe side effects of effective therapy with antimonials [66, 67], cisplatin [32] or *T*. *cordifolia* [30], many days after the end of the treatment. In contrast to what proposed previously [55], these [30–32] and our results suggest that the severe increase of liver enzymes after chemotherapy of VL with Glucantime, is due to the direct hepatotoxic effect of the drug, rather than to the killing of parasites, as serum GOT is released into blood when the liver or heart is damaged [68]. In agreement with that, Kato et al., [69] reported swollen and apoptotic hepatocytes in animals treated with Glucantime. Therefore, while all the current medications against VL are associated with hepatic toxicity [68, 70], we demonstrated that the treatment of mice with immucilins is not.

Kidney injury may also be promoted by Glucantime treatment in mice and has been discussed in human therapy [71]. Cardiotoxicity, hepatotoxicity and nephrotoxicity are the most important side effects of Glucantime [66]. The drug is rapidly excreted by the kidneys [66] but cases of renal tubular dysfunction with damage to the concentration capability of kidneys [66, 72], including acute renal failure [73, 74], have also been reported. Glucantime treatment has raised the levels of blood creatinine [66, 74, 75] and urea nitrogen [73]. Additionally, a defect in urine concentration has been attributed to the antagonist effect of antimonials on neurohypophysis hormone [66]. The hamster model is more susceptibility to *L*. *(L*.*) infantum chagasi* infection, showing pronounced increases of blood urea levels and GOT after infection. All drugs reduced urea and IA and IH were less toxic than Glucantime regarding creatinine. We conclude that IA, IH and SMIH immucillins showed no toxicity in the more resistant BALB/c mouse model, in which Glucantime was highly toxic. Furthermore, IA and IH were also less toxic than Glucantime in the more susceptible CB hamster model.

The strong efficacy of IA and IH immucillins in the hamsters model is remarkably impressive considering that, human and hamster, but not mouse macrophages, showed decreased expression of iNOS mRNA which reduces the NO production and the host response to restrict *L*. *(L*.*) donovani* replication [76, 77]. This effect is a consequence of a 100-bp subregion of the hamster iNOS promoter, which lacks a NF-IL-6 binding sequence [76, 77]. An advantage of IA and IH is their efficacy and low toxicity, even in the treatment of the hamster host with its deficient control of VL.

New chemical compounds to treat leishmaniasis would be welcomed to reduce side effects and to meet developing resistance. Therapy on BALB/c mice and CB hamsters infected with *L*. *(L*.*) infantum chagasi*, support the efficacy of immucillins IA and IH, in the control of infection with low toxicity. Our results might contribute to the development of new therapeutic protocols for the control of leishmaniasis in human and animals.
